# One-Dimensional Magnetic FeCoNi Alloy Toward Low-Frequency Electromagnetic Wave Absorption

**DOI:** 10.1007/s40820-022-00920-7

**Published:** 2022-08-20

**Authors:** Bintong Yang, Jiefeng Fang, Chunyang Xu, Hui Cao, Ruixuan Zhang, Biao Zhao, Mengqiu Huang, Xiangyu Wang, Hualiang Lv, Renchao Che

**Affiliations:** 1 Laboratory of Advanced Materials, Shanghai Key Lab of Molecular Catalysis and Innovative Materials, Department of Materials Science, Fudan University, Shanghai, 200438 People’s Republic of China; 2 Willian G. Lowrie Department of Chemical and Biomolecular Engineering, The Ohio State University, Columbus, OH 43210 USA; 3 Zhejiang Laboratory, Joint-Research Center for Computational Materials, Hangzhou, 311100 People’s Republic of China

**Keywords:** Medium-entropy magnetic alloy, One-dimension, Off-axis electronic holography technique, Improved electrospinning, Lower-frequency electromagnetic wave absorption

## Abstract

Rational designing of one-dimensional (1D) magnetic alloy to facilitate electromagnetic (EM) wave attenuation capability in low-frequency (2–6 GHz) microwave absorption field is highly desired but remains a significant challenge. In this study, a composite EM wave absorber made of a FeCoNi medium-entropy alloy embedded in a 1D carbon matrix framework is rationally designed through an improved electrospinning method. The 1D-shaped FeCoNi alloy embedded composite demonstrates the high-density and continuous magnetic network using off-axis electronic holography technique, indicating the excellent magnetic loss ability under an external EM field. Then, the in-depth analysis shows that many factors, including 1D anisotropy and intrinsic physical features of the magnetic medium-entropy alloy, primarily contribute to the enhanced EM wave absorption performance. Therefore, the fabricated EM wave absorber shows an increasing effective absorption band of 1.3 GHz in the low-frequency electromagnetic field at an ultrathin thickness of 2 mm. Thus, this study opens up a new method for the design and preparation of high-performance 1D magnetic EM absorbers.

**Supplementary Information:**

The online version contains supplementary material available at 10.1007/s40820-022-00920-7.

## Highlights

- A novel FeCoNi carbon fiber (FeCoNi/CF) is obtained through an improved electrospinning technology, which greatly endows the fiber with strong magnetic property.
- The FeCoNi/CF exhibits an enhanced electromagnetic loss capability due to the construction of one-dimensional magnetic FeCoNi alloy.
- The designed one-dimensional FeCoNi/CF exhibits excellent performance, with a broad effective absorption band of 1.3 GHz in the low-frequency electromagnetic field at an ultrathin thickness of 2 mm, which provides a great potential for practical application in the future.

**Supplementary Information:**

The online version contains supplementary material available at 10.1007/s40820-022-00920-7.

## Graphical abstract

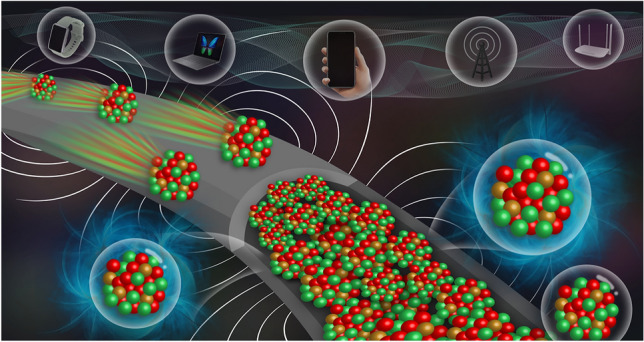

## Introduction

Continuous advancements in fifth generation (5G) wireless communication technology have catapulted the world into an intelligent era, with a plethora of wireless devices in widespread use. Despite benefiting much from 5G communication, the increased electromagnetic (EM) pollution at lower-frequency region (major concentrated at 4–6 GHz), owing to the higher emission power and lower utilization, has attracted extreme attention as compared to previous 4G, which needs to be urgently solved [1–6]. Therefore, exploring the microwave absorbers that can efficiently dissipate EM wave into Joule heat has been regarded as a feasible solution [7–9]. To obtain a desirable lower-frequency EM absorption performance, two aspects are considered: (1) component selection [10–16]; (2) structural engineering [17–23]. Concerning the component selection, while maintaining the comparable dielectric loss ability, magnetic materials show a significant magnetic loss behavior, making these materials the most promising choices [24–29]. Ferromagnetic metals have attracted more attention than other magnetic oxides among the candidates in magnetic materials due to the high magnetic loss ability resulting from the largely saturated magnetization [30–34].

However, ferromagnets comprise magnetic metals (Fe and Co etc*.*), which are easily oxidized, especially on nano- or microscale, and thus degrade EM dissipation performance over time. Even if the subsequent alloying method can slow the oxidation phenomenon to some extent, the most employed magnetic alloys generally consist of only two metals (low entropy magnetic alloy) and show poor stability [35–39]. Designing a high- or medium-entropy magnetic alloy with strong magnetic dissipation behavior is preferable to address this issue, which, unfortunately, has been largely ignored.

The effect of structure on the EM dissipation mechanism is also critical in addition to component selection [40–42]. Recently, most of the absorbing materials are zero- or three-dimensional or even have hierarchical structures. Although previous studies have not identified which structure was beneficial for magnetic or dielectric loss, the widely accepted conclusion is that compared with other dimensional structures, particularly the zero-dimensional materials, 1D structures would be a good candidate because of their the advantages of lower percolation, longer dissipation path, a higher ratio of defect sites caused by the quantum-size effect, and lower contacted resistance. Meanwhile, 1D magnetic structure would increase the magnetic anisotropy and further improve the magnetic loss processes [24, 26]. Numerous 1D materials have been investigated, such as magnetic metals, alloys or oxides [43–45]. Unfortunately, developing 1D high/medium-entropy magnetic alloys is still a challenge, which hinders the development of high-performance EM wave absorption materials.

In this work, the as-prepared FeCoNi nanoparticle (denoted as FeCoNi NP) with a diameter of 200 nm was directly encapsulated into fibers through the electrospinning process. This approach significantly enhances the fibers’ the magnetic properties, bringing the advantages of higher saturation magnetization and stronger magnetic coupling. Furthermore, the medium-entropy design upgrades the magnetic properties of the FeCoNi alloy at the atomic level, due to the improved magnetic-domain movement. Thus, the well-designed FeCoNi carbon fiber (denoted as FeCoNi/CF) exhibits outstanding EM absorption performance under a low-frequency electromagnetic field with the *f*_E_ reaching 1.3 GHz at an ultrathin thickness of 2 mm. These findings confirm the effectiveness and practicability of the FeCoNi/CF with artificially designed shape anisotropy, providing new insight into the design of 1D magnetic EM absorption materials with novel components.

## Experimental Section

### Synthesis of FeCoNi NP

FeCoNi NP was synthesized via a simple low-temperature hydrothermal process. Typically, 4 mmol FeCl_2_·4H_2_O, 3.2 mmol CoCl_2_·6H_2_O, and 0.8 mmol NiCl_2_·6H_2_O were dissolved in 30 mL deionized water and stirred for 10 min. Then, 1.5 g NaOH was dispersed in 5 mL deionized water and 15 mL N_2_H_4_·H_2_O (85 wt%) with sonication to form a mixture solution. After that, the mixture was added dropwise to the mixed salt solution prepared in the first step under vigorous stirring. When the dropping process was done, the mixture was immediately transferred into a Teflon-lined autoclave and treated at 120 °C for 12 h. Finally, the sample was magnetically separated and washed with deionized water and ethanol several times. The obtained black magnetic powders were finally vacuum dried in an oven at 60 °C overnight. FeCoNi NP with different atom ratios was synthesized with different proportions of iron salts, cobalt salts, and nickel salts.

### Synthesis of FeCoNi/CF

For the preparation of the electrospinning precursor solution, 2.6 g polyacrylonitrile (PAN) was dispersed in 14 mL DMF with magnetic stirring at 60 °C for 1 h. Then 4.8 g of as-prepared FeCoNi NP was added to the above solution, followed by mechanical stirring to ensure uniform dispersion of FeCoNi NP. After stirring for 24 h, the homogeneous precursor solution was loaded into a plastic syringe with an 18G needle. The electrospinning was conducted by applying the voltage of 14.0 kV at a collecting distance of 20 cm. The injection speed was set at 0.45 mL h^−1^. The formed carbon fiber mats were vacuum dried in an oven at 60 °C overnight. Next, the as-prepared FeCoNi/PAN fiber was pre-oxidized in the air at 240 °C for 1.5 h. And then the temperature rose to 330 °C and kept for 0, 1, and 1.5 h, respectively. Finally, the fiber was annealing in H_2_/Ar (5%) atmosphere at 700 °C for 2 h to obtain the final FeCoNi carbon fibers, which were denoted as FeCoNi/CF-1, FeCoNi/CF-2, FeCoNi/CF-3. Noticeably, all the temperature ramping rate was set to 5 °C min^−1^.

## Results and Discussion

### Synthesis and Characterization of FeCoNi/CF

**Fig. 1:**
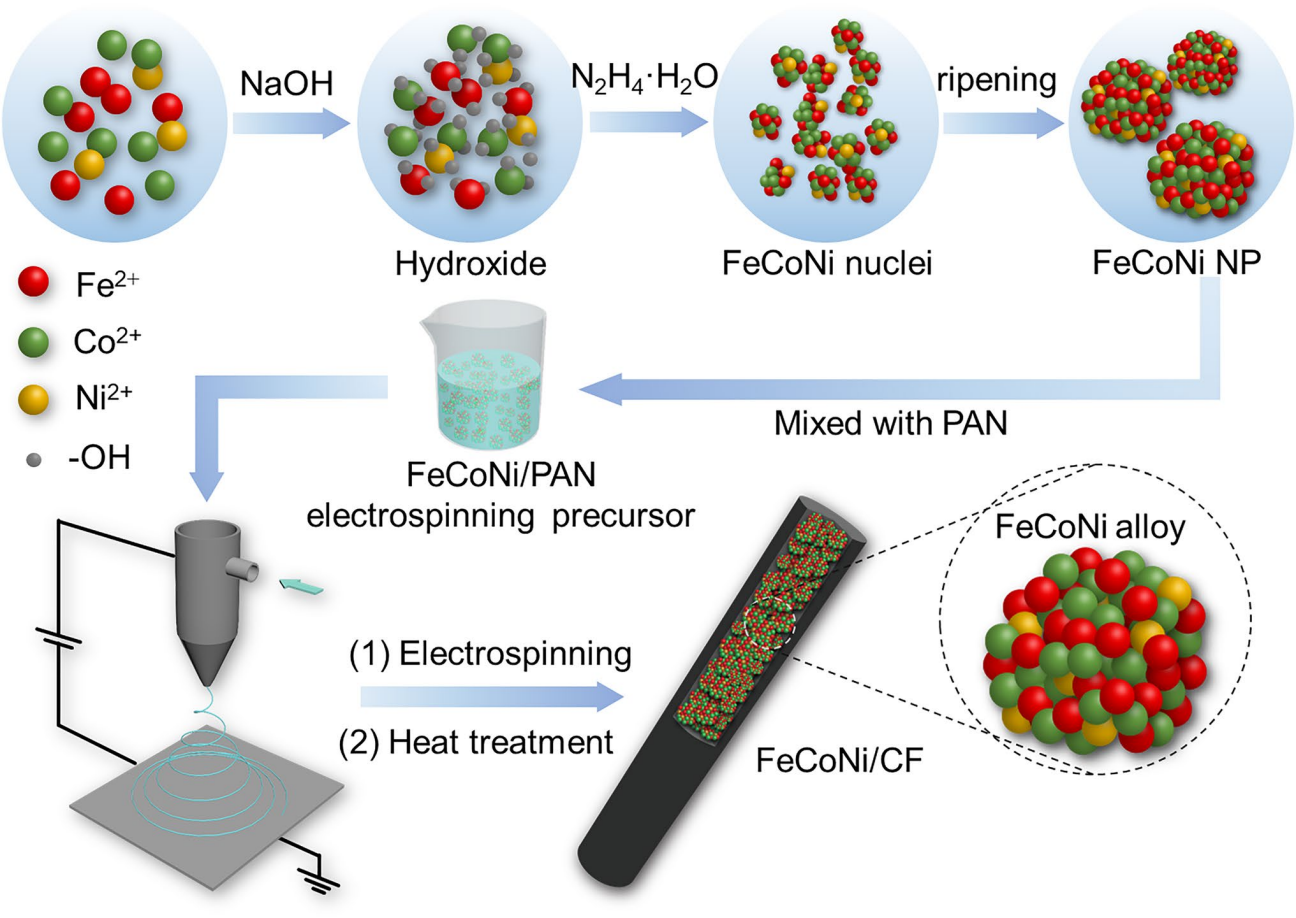
Schematic illustration of the synthetic strategy for FeCoNi/CF composites

Figure 1 shows the schematic representation of the FeCoNi/CF preparation method. First, divalent metal salt ions (Fe^2+^, Co^2+^, and Ni^2+^) combined with OH^−^ to form the hydroxide (Fe(OH)_2_, Co(OH)_2_, and Ni(OH)_2_). Then in the presence of the strong reductant hydrazine hydrate (N_2_H_4_·H_2_O), Fe^2+^, Co^2+^, and Ni^2+^ which were dynamically released from the solid hydroxide were easily reduced to metallic FeCoNi nuclei simultaneously due to the very approximate reduction potential (Fe/Fe^2+^, Co/Co^2+^, and Ni/Ni^2+^ redox pairs), atomic radius and entropy value, respectively. Then, the nuclei were gradually assembled into large FeCoNi crystals in the hydrothermal environment to reduce surface energy. The related chemical reaction equations were presented in the Supporting Information. Of particular note, excessive NaOH was required to further reduce the potential of three redox pairs based on the Nernst equation (detailed provided in Supporting Information).

Subsequently, the FeCoNi NPs were dispersed into the PAN, which was commonly used as the polymer matrix in the electrospinning process. The charged precursor droplets at the needle’s tip changed from a sphere to a cone (“Taylor cones”) under an electric field, and then the jets extended from the cone, eventually forming a PAN fiber encapsulated within FeCoNi NPs. PAN fiber underwent dehydrogenation and cyclization during the pre-oxidation process, resulting in a more stable structure. The pre-oxidation fiber was carbonized under 700 °C to form a highly conductive carbon fiber encapsulating FeCoNi after annealing in a flow of H_2_/Ar. The resulting carbon skeleton shaped the FeCoNi nanoparticles into a microchain structure. Meanwhile, it also prevented the internal magnetic particles from severe oxidation. In addition, with the pre-oxidation time increasing, the mass of the pre-oxidation fiber would decrease, affecting the carbon component in the final fiber. Therefore, the magnetic-carbon ratio in the final FeCoNi/CF could be adjusted by setting another pre-oxidation time. It was worth noting that FeCoNi nanoparticles were prepared by a hydrothermal reaction before the electrospinning process. Different from the traditional method of mixing metal ions into fibers and then reducing them under a high-temperature hydrogen atmosphere, our improved electrospinning process greatly improved the size of magnetic particles and the magnetic loading rate, which were beneficial to the formation of the strong magnetic fiber.

**Fig. 2:**
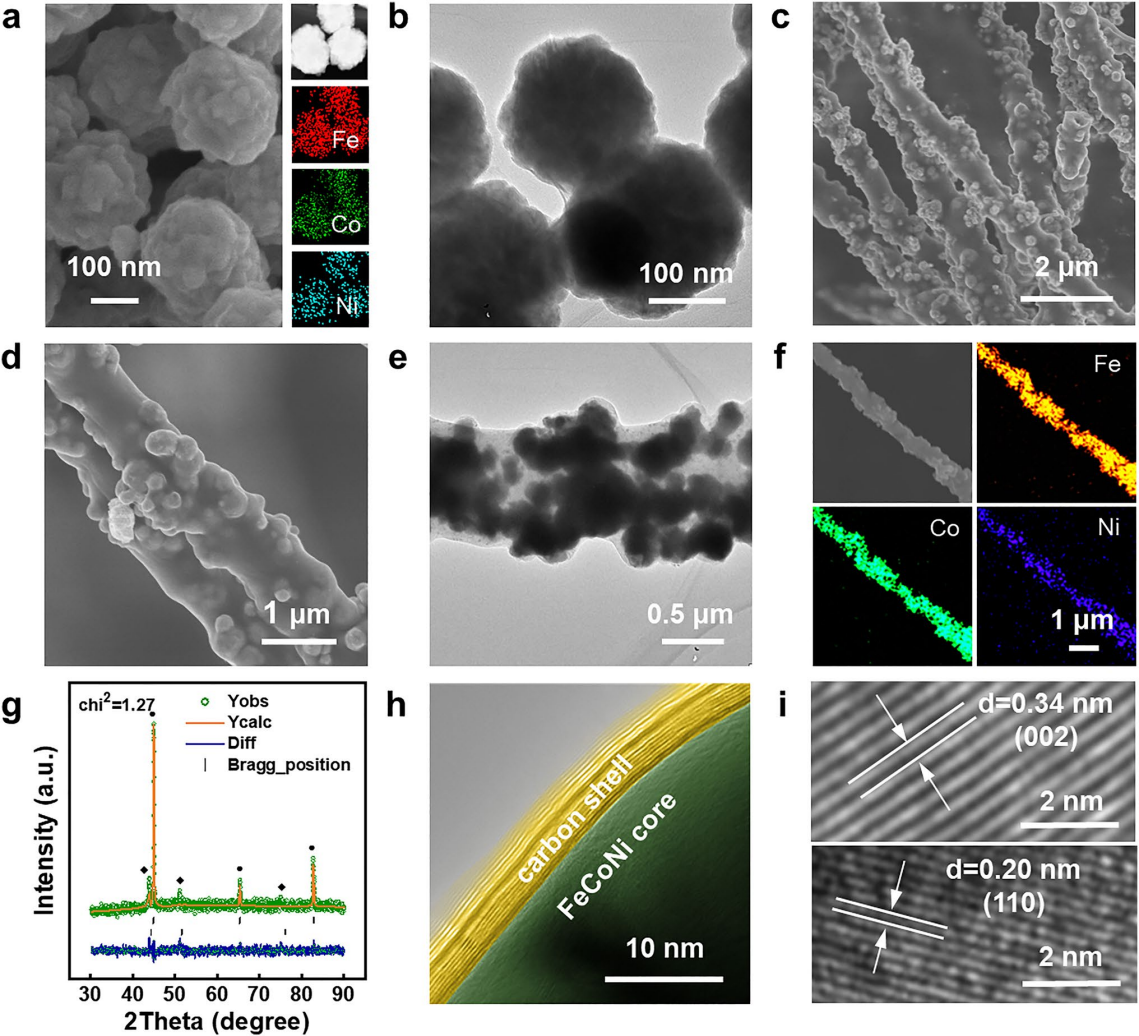
**a** SEM, EDS mapping and **b** TEM images of FeCoNi NP. **c**, **d** SEM, **e** TEM, and **f** EDS mapping images of FeCoNi/CF-1. **g** XRD Rietveld spectrum of the FeCoNi/CF-1. **h**, **i** HRTEM images of FeCoNi/CF-1

To characterize the microstructures and phase information of FeCoNi NP and FeCoNi/CF, scanning electron microscopy (SEM), transmission electron microscopy (TEM), and X-ray diffraction (XRD) were used (Fig. 2). SEM and TEM images accompanied with element mapping co-revealed that the ultimate sample showed a typically spherical structure (about 200 nm in size) and comprised Fe, Co, Ni elements uniformly (Fig. 2a–b), preliminarily proving that the FeCoNi alloy had no phase separation during the formation. Figure S1 displays the XRD pattern of FeCoNi NP which could further help analyze the phase information. The XRD pattern only exhibited three main peaks (2*θ* = 44.9°, 65.3°, and 82.7°), corresponding to the (110), (200), and (211) planes of a FeCo-type body-centered cubic structure, respectively (JCPDS No. 49-1568). Therefore, Ni atoms were considered to insert into the FeCo as interspace or displacement solution, not as split FeNi or CoNi phase.

Figure 2c–e shows the SEM and TEM images of FeCoNi/CF-1, which was comprised of a carbon fiber shell and its wrapped FeCoNi NP. The fiber skeleton had a diameter of about 1 μm, providing enough internal space for the FeCoNi NP to be tightly packed. Besides, energy-dispersive spectroscopy (EDS) was also applied to prove that FeCoNi NP was evenly distributed within the fiber, as shown in Fig. 2f. The exhibited SEM image of FeCoNi/CF-1 and corresponding elemental mapping revealed the dense and uniform distribution of Fe, Co, and Ni elements, suggesting the dense packing pattern of FeCoNi inside fibers.

Figure 2g shows the XRD Rietveld spectrum of FeCoNi/CF-1, which provided the phase information of FeCoNi after encapsulating treatment. The three most prominent diffraction peaks, (110), (200), and (211) (denoted with a black circle symbol), corresponded to FeCo-type body-centered cubic structure (JCPDS No. 49-1568). Another set of peaks was fit to the (111), (200), and (220) crystal planes of FeCoNi (denoted with a black diamond symbol). After 1D encapsulation and heat treatment, FeCoNi could still maintain the original phase. Moreover, all the characteristic peaks were sharp and clear, indicating the high purity and good crystallinity of the FeCoNi/CF sample.

HRTEM images revealed a core–shell structure with a thin graphitized carbon wrapping around the FeCoNi NP (Fig. 2h). Previous studies showed that Fe, Co, and Ni were found to be efficient catalysts for the formation of graphite encapsulated nanocrystals of magnetic metal at elevated temperature. The FeCoNi fiber consisted of localized graphitic structures and their inner magnetic particles, which enhanced the overall graphitization degree and led to an enhanced electric conductivity [46–49]. Figure 2i shows clear lattice fringes derived from the FeCoNi NP and graphitized carbon. The interplanar spacings of two randomly selected crystal lattices were 0.20 and 0.34 nm, corresponding to the (110) of FeCoNi alloy and (002) of graphitic carbon, respectively.

### EM Wave Absorption Performance and Dissipation Mechanism of FeCoNi/CF-1

**Fig. 3:**
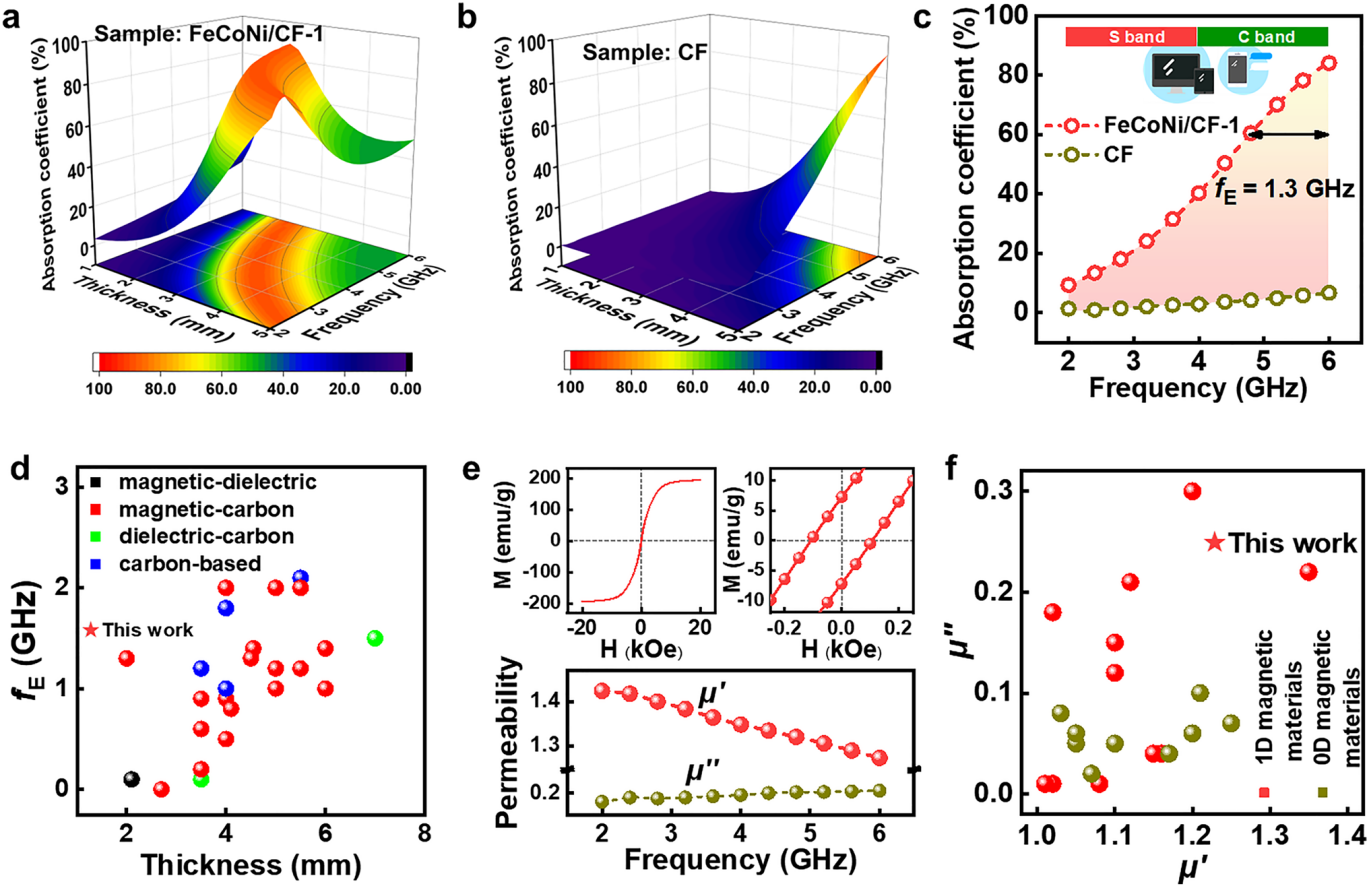
3D absorption coefficient value mapping of **a** FeCoNi/CF-1 and **b** CF as a function of thickness ranging 1–5 mm. **c** The absorption coefficient of FeCoNi/CF-1 and CF at a thickness of 2 mm. **d** Low-frequency electromagnetic absorption performance of common absorbers, as concluded according to previous research. **e** Hysteresis loops and permeability of FeCoNi/CF-1. **f** Permeability comparison of related magnetic materials, as concluded according to previous research

EM wave absorption performance of FeCoNi/CF-1 was investigated via a coaxial-line method. For comparison, we also prepared pure carbon fibers (CF) without magnetic particles. All the preparation conditions remained the same except for the absence of magnetic particles (Fig. S2). In general, the EM wave absorption performance was estimated by the reflection loss value (RL). Usually, an RL value of less than −10 dB was considered a qualified value with an absorption coefficient greater than 90%. Nevertheless, the qualified RL value in lower-frequency region (such as 2–6 GHz) was smaller than −4 dB, since it had difficulty in decoupling between impedance matching and EM wave attenuation in the lower-frequency region. Besides the RL value, the thickness was required to be smaller than 2 mm to satisfy the commercial application. Figure 3a–b shows the three-dimensional (3D) absorption coefficient value mapping of FeCoNi/CF-1 and CF as a function of thickness ranging from 1 to 5 mm. Based on the 3D absorption coefficient mapping image, we extracted the case when the absorber thickness was located in the commercial used value (2 mm), as presented in Fig. 3c. The absorption coefficient of FeCoNi/CF-1 was significantly improved within the entire 2–6 GHz band with the same filling ratio compared with CF. The effective absorption band (*f*_E_), where the RL value was greater than −4 dB, was 1.3 GHz. The absorption coefficient of CF was about zero, implying that almost no EM wave was lost. Further, to prove the low-frequency performance superiority of our as-prepared FeCoNi/CF-1, Fig. 3d and Table S1 summarize the *f*_E_ and thickness information of several common composites from previous literature. However, the low-frequency absorption performance of these materials system was non-ideal compared with our as-synthesized FeCoNi/CF-1. In addition, most materials display a narrow *f*_E_ at low frequency, or a relatively considerable *f*_E_ could only be achieved at a larger thickness. For most EM wave absorbers, this could be attributed to the poor decoupling degree between impedance matching and EM wave attenuation in the low-frequency region.

To investigate the EM wave absorption mechanism for FeCoNi/CF-1 sample in the low-frequency region, several factors could be summarized as follows: (1) designing the FeCoNi NP into the 1D carbon framework can enhance the shape anisotropy and promote the magnetic response; (2) the introduction of FeCoNi into the carbon fiber can greatly enhance the dielectric loss deriving from the enhanced electrical conductivity.

To demonstrate the former view, we used superconducting quantum interference device magnetometry and a vector network analyzer to characterize the magnetic properties of FeCoNi/CF-1 (Fig. 3e). The magnetic hysteresis loops showed that the saturation magnetization (*M*_s_) value of FeCoNi/CF-1 was 194.0 emu g^−1^, with a corresponding coercivity value (*H*_c_) of 107.4 Oe, demonstrating its desirable soft ferromagnetic nature. The relationship between dissipation ability (*μ*′′) and saturation magnetization (*M*_s_) is expressed by the following formula:

1 $$\mu_{i} = \frac{{M_{s}^{2} }}{{akH_{c} M_{s} + b\lambda \xi }}$$

where *λ* and* ξ* are the magnetostrictive coefficient and elastic strain parameter, respectively. Constant *a* and *b* are determined by the composition of the material itself, and *k* represents the proportional coefficient. The formula suggested that a large static saturation magnetization and a small coercive force can contribute to higher magnetic permeability, improving the material’s dynamic magnetic loss performance in an altered electromagnetic field. For FeCoNi/CF-1, the average level of *μ*′′ was 1.4, while the imaginary part was above 0.2. However, CF had no magnetic components, and the real and imaginary parts of the permeability were 1 and 0, respectively, which was unable to provide dynamic magnetic loss (Fig. S3).

Generally, the magnetic loss of metallic composites can be derived from four different scales, including domain wall resonance, natural ferromagnetic resonance, eddy current, and hysteresis loss. The hysteresis loss is neglectable because it can only occur at extremely high external magnetic fields. The domain wall resonance, including domain wall displacement and domain wall rotation, occurred near its intrinsic resonant frequency, which was usually in an ultra-low frequency (MHz level). Eddy current loss was usually considered to dominate in the high-frequency region (8–18 GHz), because the calculated eddy current loss factor (C_o_ = *μ''*(*μ'*)^−2^*f*^−1^) remained a constant in this region. At lower frequencies, natural ferromagnetic resonance dissipation was caused by the inherent magnetic interactions of different parts within a particle, which were coupled together to form a complex magnetic dissipation network. For ferromagnetic crystals, owing to the existence of magnetocrystal anisotropic equivalent field, natural resonance occurred under the action of an external alternating magnetic field, which was also the main source of our magnetic loss. To better demonstrate that the magnetic properties of the fibers can be greatly enhanced using our improved electrospinning technique, the permeability of our as-prepared material was compared with those in previous literature (Fig. 3f and Table S2). It can be seen that the *μ*′ and *μ*′′ of most material in the previous research would not exceed 1.3 and 0.2. Through our improved electrospinning process, the values of *μ*′ and *μ*′′ were simultaneously promoted, which was beneficial to decoupling between impedance and EM wave loss. Through comparing our material with those of magnetic materials in some literature, the magnetic superiority of our as-synthesized 1D FeCoNi/CF was further confirmed.

**Fig. 4:**
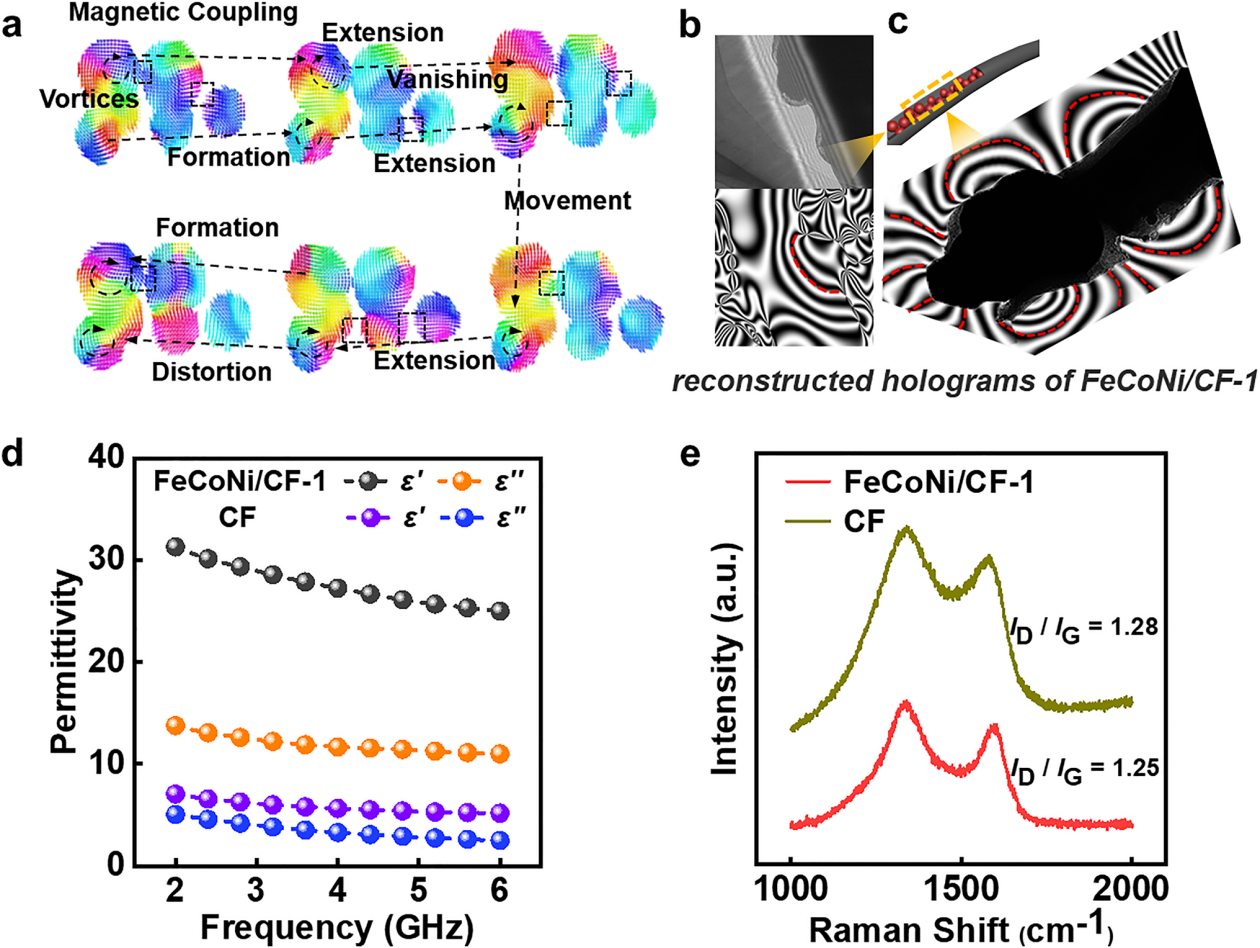
**a** Micromagnetic simulation of 1D axially distributed FeCoNi NP (The external magnetic field frequency is 6 GHz, and the diameter of the microsphere is 200 nm). **b** Electron holograms of FeCoNi/CF-1 and corresponding stray magnetic field distribution. **c** TEM image of FeCoNi/CF-1 and the corresponding stray magnetic field distribution with recombination of different regions. **d** Permittivity of FeCoNi/CF-1 and CF. **e** Raman spectra of FeCoNi/CF-1 and CF

To further shed on the magnetic loss mechanism, the micromagnetic simulation was used to explore the magnetic loss lifting mechanism of FeCoNi/CF-1. Here a cylindrical model with FeCoNi NP randomly dispersed was constructed (Fig. 4a). A typical dynamic evolution process of magnetic vortices was illustrated under different magnetic fields, including extension, distortion, vanishing, formation, and movement. Such coupling interactions vanished and reappeared frequently between two adjacent particles, resulting in a dramatic variation in the interior magnetic moment. The sample’s frequent variation could cause domain walls to migrate, resulting in magnetic loss [21, 22]. Extra energy from external fields was required to rebuild the dynamic magnetic network during this construction and destruction process of the magnetic coupling network, resulting in a phase lag between internal magnetic moments and external fields. Besides, the magnetic moments marked with black dotted lines appeared parallel or opposite, proving the existence of magnetic coupling. Consequently, the unique 1D structure considerably boosted the magnetic loss capacity. The use of high-density magnetic lines and coupling networks aided the enhancement of magnetic loss, resulting in better EM wave absorption performance.

Further, off-axis electronic holography was first-time used to evaluate the magnetic property of the as-synthesized FeCoNi/CF (Fig. 4b–c). The reconstructed hologram showed that the magnetic field lines radiated from one particle to the other, forming a closed loop between two adjacent magnetic particles, demonstrating magnetic coupling. The fibers exhibited integral magnetic line distribution rather than disarray distributions, implying compulsory assembly magnetic interactions due to the dense packing of inner FeCoNi NP. Magnetic dissipation was further aided by the high-density and wide range of magnetic coupling networks, resulting in improved EM wave absorption performance.

Next, we discussed the effect on the electrical conductivity after introducing FeCoNi into the fiber. In addition, the insertion of FeCoNi alloy also attributed to the dielectric loss, as discussed as follows: As shown in Fig. 4d, the pure CF showed the lower permittivity value, with both real and imaginary parts below 10, which was due to the poor graphitization degree. As a result, we observed the poor EM absorption performance for the pure CF. After inserting the conductive FeCoNi into CF, significant enhancement could be observed for the FeCoNi/CF material system. For example, the permittivity was greatly improved for the FeCoNi/CF-1 sample, with the real part enlarged to 5 times and imaginary part to 3 times. Raman spectroscopy was used to detect the degree of graphitization of the carbon fiber (Fig. 4e). D and G peaks were the Raman characteristic peaks of C atom crystal, which were located near 1320 and 1585 cm^−1^, respectively, representing the crystal defects of C atom and the in-plane stretching vibration of C atom *sp*^2^ hybrid, respectively. The *I*_D_/*I*_G_ value of FeCoNi/CF-1 decreased to 1.25, compared with CF (1.28), due to the increased G peak value which was derived from the excellent graphite catalytic ability of FeCoNi. A lower *I*_D_/*I*_G_ value can result in a greater initial dielectric value, which can improve the low-frequency EM wave absorption performance [49].

### Performance of Other FeCoNi/CF Samples

**Fig. 5:**
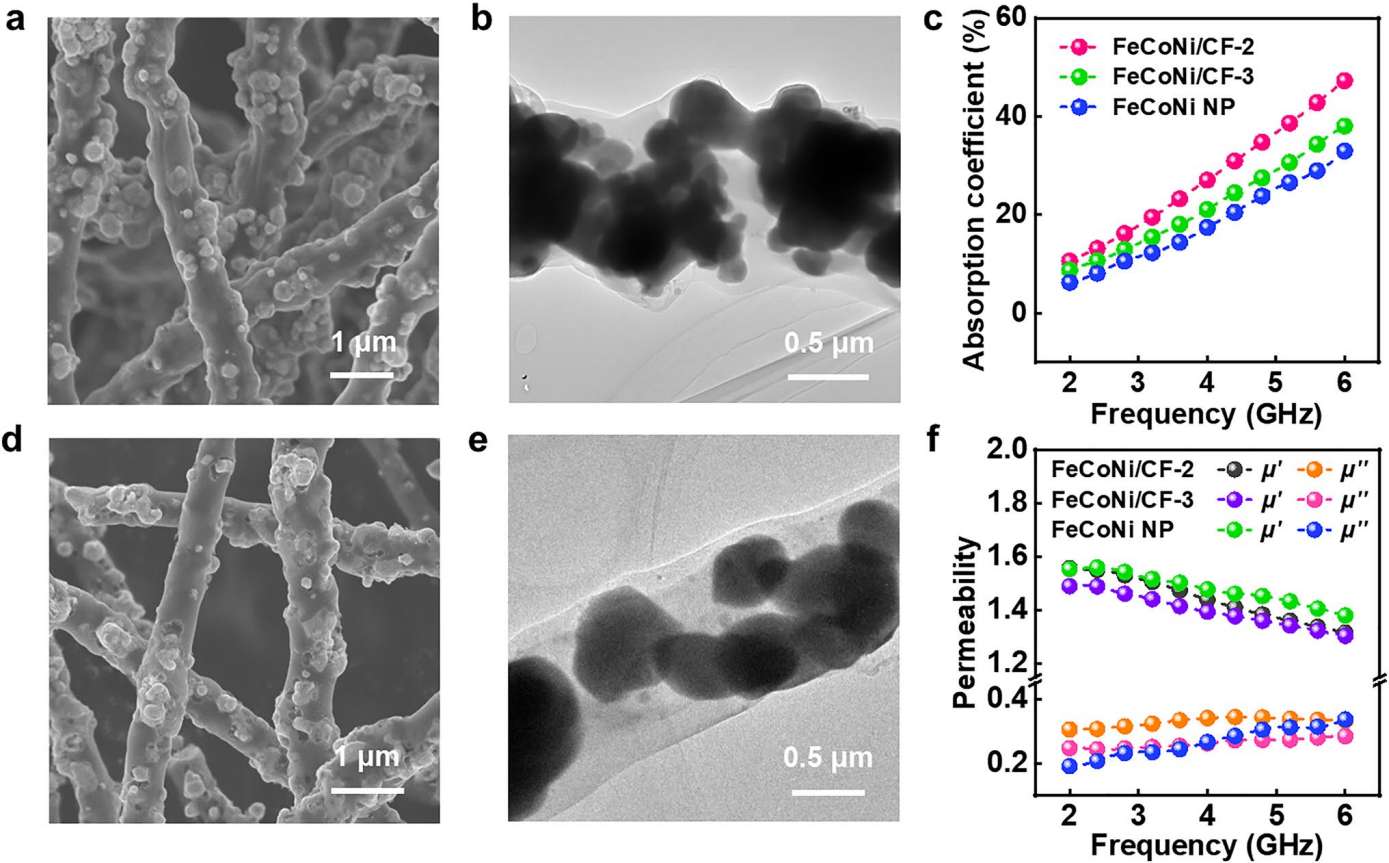
**a** SEM and **b** TEM images of FeCoNi/CF-2. **c** The absorption coefficient of FeCoNi/CF-2, FeCoNi/CF-3 and FeCoNi NP at thickness of 2 mm. **d** SEM and **e** TEM images of FeCoNi/CF-3. **f** Permeability of FeCoNi/CF-2, FeCoNi/CF-3 and FeCoNi NP

To demonstrate the universality of the excellent absorbing properties of as-prepared 1D magnetic fibers, we also synthesized FeCoNi/CF with different magnetic-carbon component ratios (denoted as FeCoNi/CF-2 and FeCoNi/CF-3). Based on sample FeCoNi/CF-1, sample FeCoNi/CF-2 and FeCoNi/CF-3 added another pre-oxidation process stage, which aimed to regulate the magnetic-carbon ratio in the final formed FeCoNi/CF by controlling the mass of pre-oxidation FeCoNi. Figure 5a–b and d–e shows the SEM and TEM images of FeCoNi/CF-2 and FeCoNi/CF-3, respectively, which exhibited that FeCoNi/CF-2 and FeCoNi/CF-3 had 1D fibrous structures encapsulated with FeCoNi magnetic particles. The TEM image revealed that the agglomeration of magnetic nanoparticles increased from FeCoNi-2 to FeCoNi-3 as the heating treatment time increased. The XRD patterns of FeCoNi/CF-2 and FeCoNi/CF-3 exhibited similar peak shape as FeCoNi/CF-1 (Fig. S4). It showed that the phase of the sample main components can still be maintained under the variable condition, proving the stability of the structure.

Figure S5 shows the 3D absorption coefficient value of FeCoNi/CF-2, and FeCoNi/CF-3 within 2–6 GHz as a function of thickness ranging from 1 to 5 mm. Notably, the EM wave absorption performance of FeCoNi NP was also displayed to prove the superiority of 1D magnetic fiber. We still extracted the thickness under 2 mm and depicted it in Fig. 5c. The absorption coefficient of FeCoNi/CF-2 and FeCoNi/CF-3 was significantly improved compared with that of CF and FeCoNi NP, which can be attributed to the template construction of 1D magnetic anisotropic fibers. The magnetic permeability of FeCoNi/CF-2 and FeCoNi/CF-3 exhibited a slightly higher value compared to FeCoNi/CF-1 (Fig. 5f), which might be attributed to the higher magnetic-carbon ratio. The imaginary part of the magnetic permeability of FeCoNi/CF was much higher than that of FeCoNi NP, suggesting the superiority of the 1D structure. We tested the hysteresis loop of the above samples and discovered that they had high static saturation magnetization and good soft magnetic properties, all of which resulted in excellent permeability (Fig. S6). The permittivity is also listed in Fig. S7 which showed a decreased trend when the carbon content of FeCoNi/CF decreased, as the permittivity and conductivity were positively correlated. In addition, when the pre-oxidation time continued to extend, the carbon content in the fiber continued to decrease, which would cause the dielectric constant to drop to a lower level. A lower dielectric constant was not conducive to the generation of excellent absorbing properties. Therefore, the pre-oxidation time cannot be extended indefinitely. From the perspective of dimension construction of FeCoNi alloy, it was found that the 1D form had a great improvement in both permittivity and permeability, which further proved the advantage of 1D FeCoNi alloy.

### Component Selection Engineering of FeCoNi/CF

**Fig. 6:**
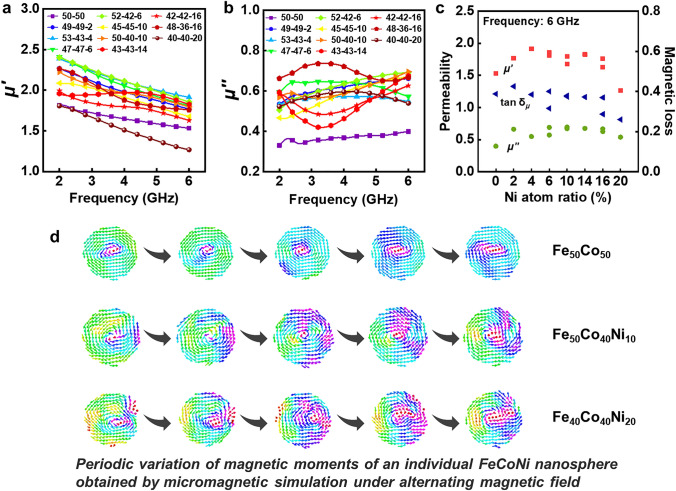
**a** *μ*′ and **b** *μ*′′ of FeCoNi NP of different Fe, Co, Ni ratios. **c** The permeability and magnetic loss values of FeCoNi NP with different Ni atom ratios under 6 GHz. **d** Micromagnetic simulation of FeCoNi spheres with cyclical variation in different Fe/Co/Ni ratios. The external magnetic field frequency is 6 GHz, and the diameter of the microsphere is 200 nm

Besides structure engineering, the component selection was also utilized in this work. Generally, upgrading the magnetic loss capacity intrinsically was essential. The magnetic property of FeCoNi/CF was closely linked to its complex permeability. Doping FeCo alloy with heterogeneous Ni elements can considerably improve magnetic loss and storage ability. Thus, electromagnetic parameters of 11 kinds of FeCoNi NP with different proportions of Fe, Co, and Ni elements were measured to validate this hypothesis (Fig. 6a–b). ICP analysis was applied to identify the atomic ratio of Fe, Co, and Ni in different samples. The results showed that FeCoNi with different element ratios had different *μ′* and *μ′′* values, corresponding to different intrinsic magnetic loss capabilities. We extracted magnetic parameters and Ni content data at 6 GHz and discovered that the magnetic parameters reached a maximum range when Ni content was in the middle range (4%–14%) (Fig. 6c). This implied that adding Ni into FeCo could improve intrinsic magnetism of the material, which was obtained via ferromagnetic resonance and natural loss, making a significant effect on the loss of electromagnetic waves. Furthermore, larger magnetic parameters were beneficial to stronger absorption performance under thinner thickness, according to the calculation formula of reflection loss.

The micromagnetic simulation was used to explain this phenomenon. Figure 6d shows the simulated dynamic magnetic response of individual FeCoNi microsphere with three representative ratios (Fe_50_Co_50_, Fe_50_Co_40_Ni_10_, and Fe_40_Co_40_Ni_20_). The magnetic moments at the edge regions vibrated more violently during a single period as the Ni content increased, indicating more intensive energy dissipation. Moreover, the Ni-rich particles showed a broader magnetic-domain vibration region, demonstrating a broader response to the alternating field. Such dynamic domain movement can cause significant energy loss when interacting with an electromagnetic field, resulting in an enhanced magnetic loss.

FeCoNi/CF was an excellent magnetic-dielectric microwave absorber, covering both magnetic loss and dielectric loss, and the former dominated at low frequency. The coupling degree between input impedance and EM wave dissipation capability increased significantly as frequency decreased, which would result in a poor EM wave absorption property. To solve the coupling issue between input impedance and EM wave dissipation, magnetic materials with excellent magnetic constant can regulate the input impedance and EM wave dissipation ability greatly and thus exhibit superior EM wave absorption performance in the low-frequency region. Though structural design or component regulating, higher magnetization which leads to enhanced permeability was achieved for our as-prepared FeCoNi/CF.

## Conclusion

In summary, this study provides a unique FeCoNi alloy confined in directionally extended CF, which allows the enhancement of magnetic response and magnetic shape anisotropy. Moreover, the FeCoNi alloy has enhanced magnetic loss capacity due to the novel multielement metal alloying method. In addition, the decorated 1D carbon fibers form a 3D conductive network in space, which contributes to the conductive loss. Notably, such a 1D shape can be further applied to other magnetic structures which require good shape anisotropy. In a nutshell, this unique magnetic fiber can inspire new designs of 1D magnetic EM wave absorption materials.

## Supplementary Information

Below is the link to the electronic supplementary material.

Supplementary file1 (PDF 599 KB)
