# Supplementary material for: One-Dimensional Magnetic FeCoNi Alloy Toward Low-Frequency Electromagnetic Wave Absorption
Source: Nanomicro Lett. 2022 Aug 20;14:170. doi: 10.1007/s40820-022-00920-7 (PMC9392832; doi:10.1007/s40820-022-00920-7)
Supplement: Supplementary file 1 — Supplementary file1 (PDF 599 KB) [file 40820_2022_920_MOESM1_ESM.pdf]

Supporting Information for

One-Dimensional Magnetic FeCoNi Alloy Toward Low-Frequency Electromagnetic Wave Absorption

Bintong Yang[1], Jiefeng Fang[1], Chunyang Xu[1], Hui Cao[1, ∗] , Ruixuan Zhang[1], Biao Zhao[1], Mengqiu Huang[1], Xiangyu Wang[1], Hualiang Lv[2, ∗], Renchao Che[1, 3, ∗]

[1]Laboratory of Advanced Materials, Shanghai Key Lab of Molecular Catalysis and Innovative Materials, Department of Materials Science, Fudan University, Shanghai 200438, P. R. China

[2]Willian G. Lowrie Department of Chemical and Biomolecular Engineering, The Ohio State University, Columbus, OH, 43210, USA

[3]Joint-Research Center for Computational Materials, Zhejiang Laboratory, Hangzhou 311100, P. R. China

∗Corresponding authors. E-mail: yexuexun5309@163.com (Hualiang Lv), caohui@fudan.edu.cn (Hui Cao), rcche@fudan.edu.cn (Renchao Che)

S1 Micromagnetic Simulation Methods

The micromagnetic simulation is executed by Mumax3 software, which is an open-source GPU accelerated program. It calculates the space- and time-dependent magnetization dynamics in nano- to micro-sized ferromagnets using a finite-difference discretization. According to the Landau-Lifshitz Gilbert equation and the principle of minimum energy as well as the finite difference algorithm, the dynamic physical process stimulation and 3D model solving process are carried out.

In this work, the simulation program is carried on to simulate the dynamic spin structure of an individual FeCoNi sphere with a tuning component and several FeCoNi spheres distributed in different ways. For the FeCoNi sphere, the diameter of the sphere is 200 nm.

The magnetic parameters of the FeCoNi spheres are calculated by a weighted average method. The saturation magnetization of Fe, Co and Ni are M_s = 1.2×10^6, 1.4×10^6, and 4.9×10^5 A m^{-1}, respectively. The micromagnetic exchange constant of Fe, Co and Ni are A = 1.7×10^{-11}, 2.1×10^{-11}, and 9×10^{-12} J m^{-1}, respectively. The magnetocrystalline anisotropy constant of Fe and Co are 5.2×10^4 and 2.65×10^5 J m^{-3}. The damping factor of spin precession is 0.01. The attached software Muview is applied to visualize the results calculated by Mumax. And the external magnetic field frequency is 6 GHz.

S2 Calculation of Reduction Potentials of Fe^{2+}/Fe, Co^{2+}/Co and Ni^{2+}/Ni Redox Pairs under Excessive NaOH

The reduction potentials of Fe^{2+}/Fe, Co^{2+}/Co and Ni^{2+}/Ni redox pairs for this typical process can be calculated using Nernst equation based on solubility product constant (K_{sp}) of solid hydroxide. The standard reduction potentials are

$$\Phi^0(Fe^{2+}/Fe) = -0.41\ V,\ \Phi^0(Co^{2+}/Co) = -0.29\ V,\ \Phi^0(Ni^{2+}/Ni) = -0.25\ V$$

Values of K_{sp} are

$$K_{sp}(Fe(OH)_2) = 7.9\times10^{-15},\ K_{sp}(Co(OH)_2) = 2.5\times10^{-16},\ K_{sp}(Ni(OH)_2) = 6.5\times10^{-18},$$

The concentration of original OH^- is 0.75 M, while 0.32 M is reacted with Fe^{2+}, Co^{2+} and Ni^{2+}. So the concentration of free OH^- is

$$[OH^-] = (0.75-0.32) = 0.43\ M$$

The concentration of metal ions in the presence of 0.75 M NaOH are

$$[Fe^{2+}] = \frac{K_{sp}[Fe(OH)_2]}{[OH^-]^2} = 4.27\times10^{-14}$$

$$[Co^{2+}] = \frac{K_{sp}[Co(OH)_2]}{[OH^-]^2} = 1.35\times10^{-15}$$

$$[Ni^{2+}] = \frac{K_{sp}[Ni(OH)_2]}{[OH^-]^2} = 3.52\times10^{-17}$$

Therefore, reduction potentials by Nernst equation are given as:

$$\Phi(Fe^{2+}/Fe) = \Phi^0(Fe^{2+}/Fe) + \frac{RT}{2F}\ln[Fe^{2+}] = -0.79V$$

$$\Phi(Co^{2+}/Co) = \Phi^0(Co^{2+}/Co) + \frac{RT}{2F}\ln[Co^{2+}] = -0.73V$$

$$\Phi(Ni^{2+}/Ni) = \Phi^0(Ni^{2+}/Ni) + \frac{RT}{2F}\ln[Ni^{2+}] = -0.73V$$

Where R is the universal gas constant: R = 8.314 J· K^{-1}· mol^{-1} and F is the Faraday constant: F = 9.649$\times$10^4 C mol^{-1}. T is room temperature: T = 293.15 K.

Fe^{2+}, Co^{2+} and Ni^{2+} released from hydroxide are simultaneously reduced to metallic atoms, which spontaneously converted to FeCoNi alloy nuclei due to closer value of reduction potential in the presence of $N_2H_4\cdot H_2O$ and excessive NaOH.

The relevant reactions could be formulated as:

$$Fe^{2+} + Co^{2+} + Ni^{2+} + NaOH \rightarrow Fe(OH)_2 + Co(OH)_2 + Ni(OH)_2 + Na^- + H_2O$$

$$Fe(OH)_2 \leftrightarrow Fe^{2+} + 2OH^-$$

$$Co(OH)_2 \leftrightarrow Co^{2+} + 2OH^-$$

$$Ni(OH)_2 \leftrightarrow Ni^{2+} + 2OH^-$$

$$Fe^{2+} + Co^{2+} + Ni^{2+} + N_2H_4\cdot H_2O + OH^- \rightarrow FeCoNi + N_2 + H_2O$$

S3 Synthesis Process of CF

For the preparation of carbon fibers (CF), 2.6 g polyacrylonitrile (PAN) was dispersed in 14 mL DMF with magnetic stirring at 60 °C for 1 h. To maintain the same fiber diameter as FeCoNi/CF, the electrospinning process was carried out at a high positive voltage (12.0 kV) and a collecting distance of 15 cm. The rest process remained the same.

S4 Characterization

The phase information of the as-synthesized samples was recorded using the X-ray diffractometer (XRD, Bruker D8 ADVANCE) with CuK_α radiation (λ=0.15406 nm). The morphology, microstructure and size of the samples were characterized by scanning electron microscopy (SEM, Hitachi S-4800, Japan) and transmission electron microscopy (TEM, JEOL JEM-2100F, Japan, 200 kV). The hysteresis loops of the as-prepared magnetic samples were measured by superconducting quantum interference device (SQUID) magnetometry (MPMS VSM, Quantum Design Company). The Raman spectra were tested with a Renishaw Invia

spectrometer using a 514 nm laser excitation. The electromagnetic parameters (complex permittivity and permeability) within 2−18 GHz were tested by vector network analyzer (Agilent N5230C). The as-prepared sample and paraffine were mixed in a ratio of 1:1 and then compressed into a columnar ring (Φ_{inner} = 3 mm, Φ_{outer} = 7 mm) by the coaxial waveguide cavity. The RL was a key indicator for the evaluation of EM absorption performance, which was calculated on the basis of the transmission line theory with equations as follows:

$$Z_{\mathrm{in}} = \sqrt{\frac{\mu_{\mathrm{r}}}{\varepsilon_{\mathrm{r}}}} \tanh\left| -j \frac{2\pi fd}{c} \sqrt{\varepsilon_{\mathrm{r}}\mu_{\mathrm{r}}} \right|$$

$$RL\ (dB) = -20\log\left| \frac{Z_{\mathrm{in}} - 1}{Z_{\mathrm{in}} + 1} \right|$$

where ε_r ($\varepsilon_r = \varepsilon' - j\varepsilon''$) and μ_r ($\mu_r = \mu' - j\mu''$) are the relative complex permittivity and permeability. f is the frequency. d is the thickness. c is the velocity of light in free space. And Z_{in} is the normalized input impedance of a metal-backed microwave absorbing layer. RL represents the reflection loss.

S5 Electron Holography Technology

The off-axis electron holography technology was used to characterize the magnetic properties of FeCoNi/CF in the nano-micrometer range. The experiment was performed with a specially designed JEM-2100F transmission electron microscope. Electron holography is able to analyze the electron wave phase because it splits the electron beam into an object wave electron beam that passes through the sample and a reference wave electron beam that travels through a vacuum. A double prism is placed behind the magnetic lens. After applying an electric field to the double prism, the two electron beams are deflected together and interfere on the imaging plane. The phase of the object wave electron beam changes after passing through the sample, due to its internal or external electric field. Based on our as-prepared sample, this phase change can be used to deconstruct stray magnetic field information at the periphery of electromagnetic functional materials.

S6 Supplementary Figures and Tables

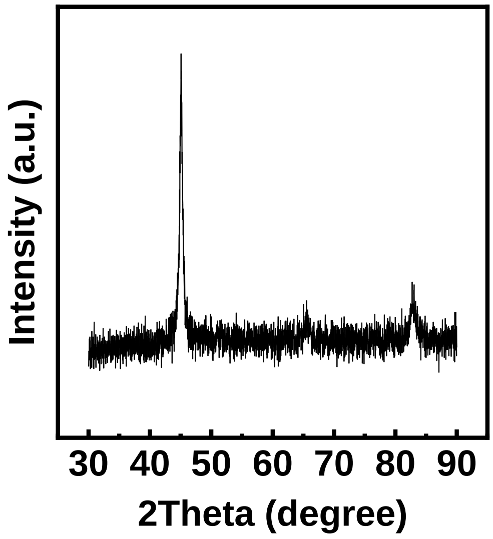

Fig. S1 XRD pattern of FeCoNi NP

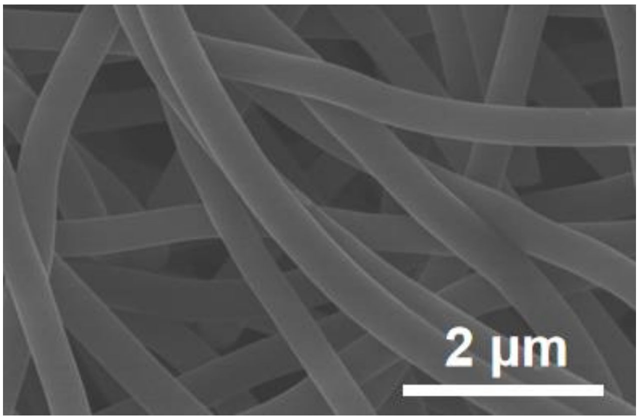

Fig. S2 The SEM image of CF

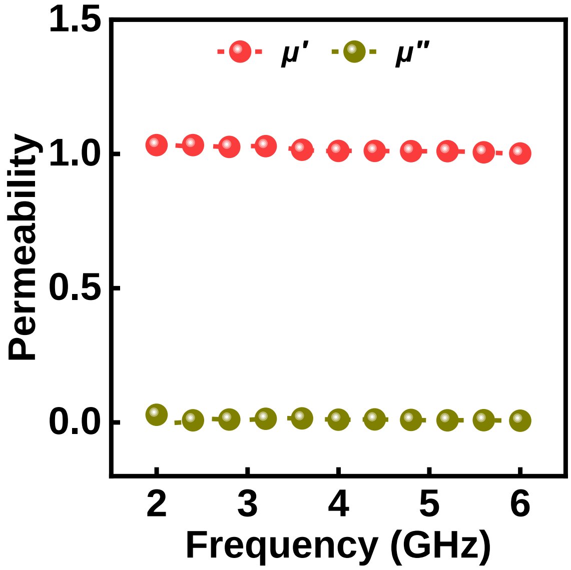

Fig. S3 The permeability of CF

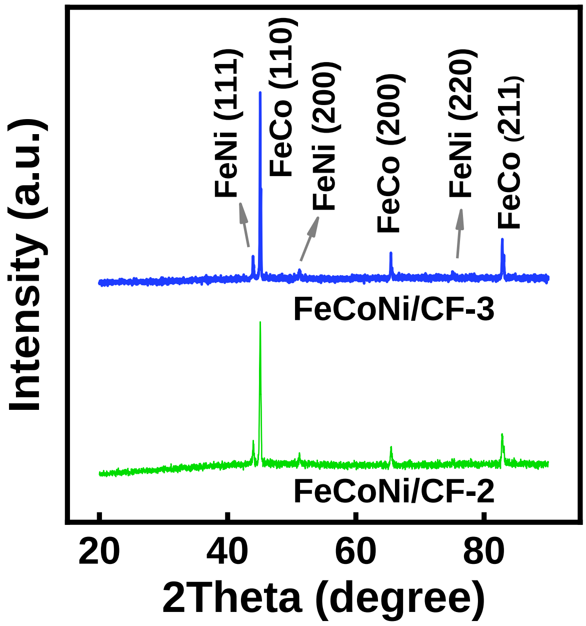

Fig. S4 The XRD patterns of FeCoNi/CF-2 and FeCoNi/CF-3

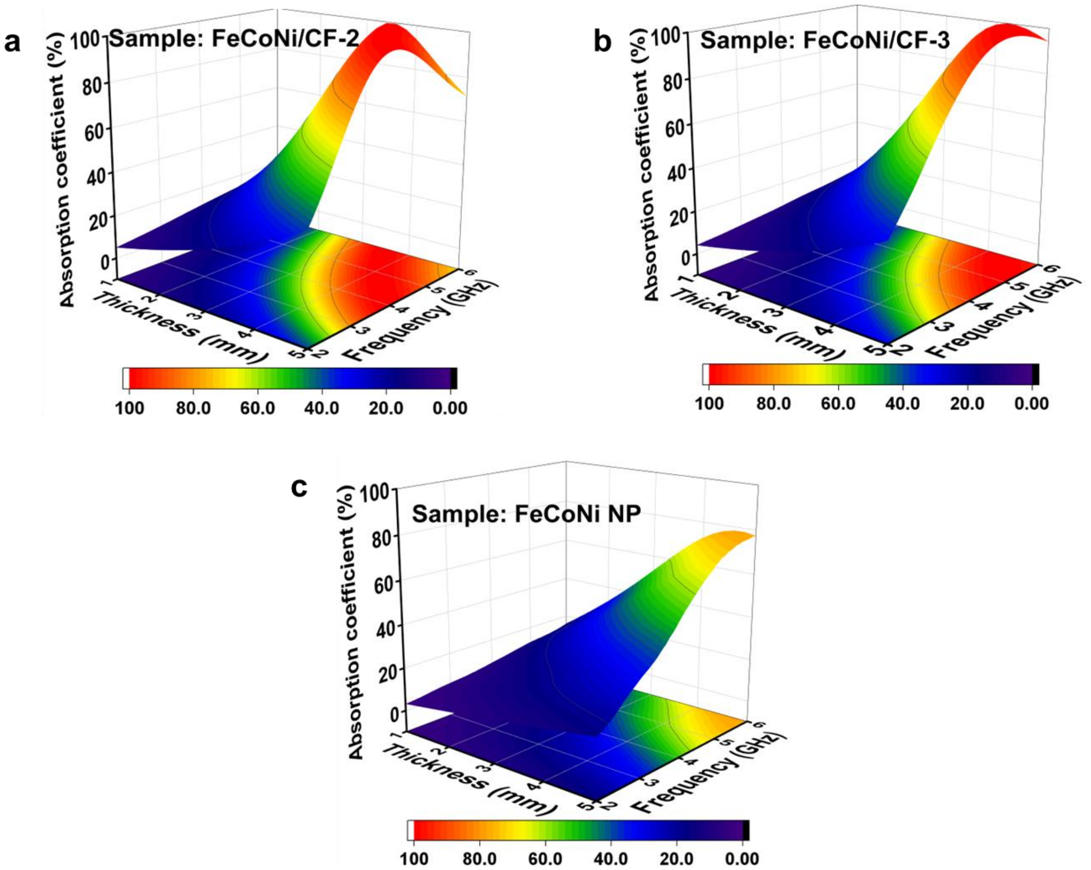

Fig. S5 The 3D absorption coefficient value mapping of **a** FeCoNi/CF-2, **b** FeCoNi/CF-2 and **c** FeCoNi NP as a function of thickness ranging 1-5 mm

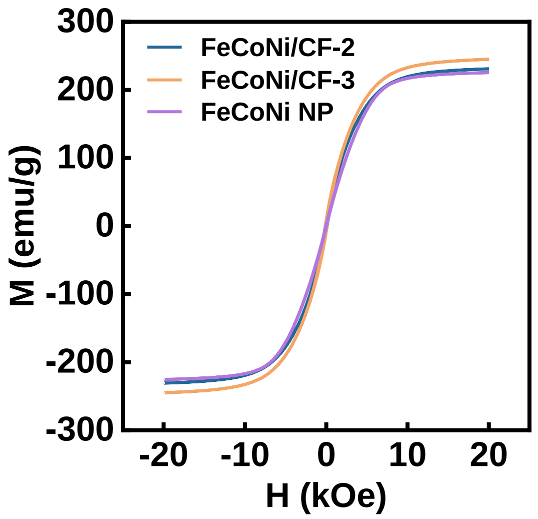

Fig. S6 Hysteresis loops of FeCoNi/CF-2, FeCoNi/CF-3 and FeCoNi NP

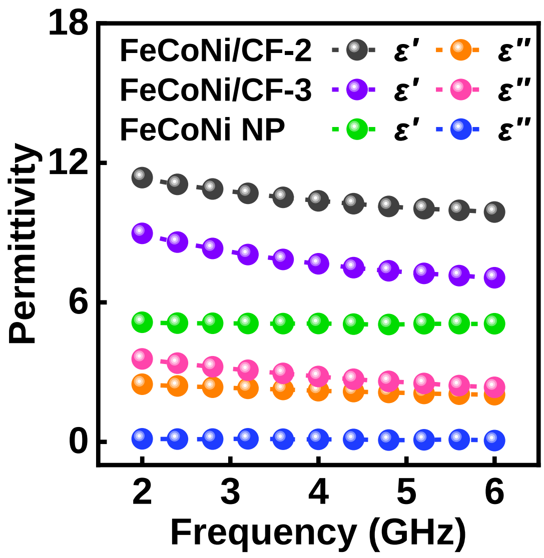

Fig. S7 Permittivity of FeCoNi/CF-2, FeCoNi/CF-3 and FeCoNi NP

Table S1 Low-frequency electromagnetic absorption performance of some common absorbers

**Absorber**	***d* (mm)**	**f_E (GHz)**
CoNi@SiO_2@TiO_2 [S1]	2.1	0.1
nitrogen-doped porous carbon composites [S2]	5.5	1.2
MnO@carbon nanowires [S3]	3.5	0.2
3D bio-carbon foams [S4]	3.5	1.2
Fe_3O_4@C nanorods [S5]	4	1.0
porous carbon nanoparticles [S6]	4	1.8
FeNi/C nanofibers [S7]	2.7	0
Fe/C porous nanofibers [S8]	3.5	0.9
graphene-based aerogel microspheres [S9]	4	1
Co/C nanofibers [S10]	6	1.4
ZnO/Co hybrid nanotubes [S11]	4.1	0.8
porous carbon nanofibers and rGO [S12]	5.5	2.1
C–$NiCo_2O_4$ nanofibers [S13]	5	2
FeCo@C-PCFs [S14]	4.55	1.4
Co_3O_4/carbon membrane [S15]	3.5	0.2
MnO-VN/C nanofibers [S16]	7	1.5
Fe_3C/C nanofibers [S17]	3.5	0.6
FeCo nanofibers [S18]	4.5	1.3
carbon/Fe_3C nanofibers [S18]	5	1
Co-C nanofibers [S19]	5	1.2
C/Co nanofibers [S20]	4	2
$NixZn_{(1-x)}Fe_2O_4$ ferrite nanofibers [S21]	4	0.5
$CoFe_2O_4$ nanofibers [S22]	6	1
TiO_2@Co/C@Co/Ni multilayered microtubes [S23]	5.5	2
FeCoNi/CF (this work)	2	1.3

Table S2 Permeability comparison of related magnetic materials

Absorber	μ'	μ''
Fe_3O_4@$Ti_3C_2T_x$/CNTs [S24]	1.1	0.12
Fe_3O_4 nanowire [S25]	1.2	0.3
$ZnFe_2O_4$ nanofibers [S26]	1.12	0.21
Fe/SiC [S27]	1.16	0.04
Fe_3C/N-doped carbon fibers [S28]	1.08	0.01
ZnFe/Fe_3C@C porous nanofibers [S29]	1.02	0.01
TiO_2/Co/carbon nanofibers [S30]	1.01	0.01
CNT/Co/C fiber [S31]	1.15	0.04
Co/N-C NFs [S10]	1.05	0.06
Co_3O_4/N-doped CF [S32]	1.1	0.15
C–$NiCo_2O_4$ nanofibers [S13]	1.02	0.18
FeCo/C [S33]	1.17	0.04
$CoFe_2O_4$/graphene [S34]	1.21	0.1
Co-carbon ball [S35]	1.03	0.08
CoFe@C [S36]	1.1	0.05
FeCo alloy nanoparticles [S37]	1.07	0.02
C/(C@CoFe) [S38]	1.05	0.05
rGO/$CoFe_2O_4$/FeCo [S39]	1.2	0.06
$CoFe_2$/$BaTiO_3$ [S40]	1.25	0.07
HCF@NC/Co [S41]	1.05	0.06
FeCoNi/CF (this work)	1.35	0.22

Supplementary References

[S1] Q. Liu, Q. Cao, H. Bi, C. Liang, K. Yuan et al., CoNi@SiO_2@TiO_2 and CoNi@Air@TiO_2 Microspheres with strong wideband microwave absorption. Adv. Mater. **28**(3), 486-490 (2016). https://doi.org/10.1002/adma.201503149

[S2] Q. Wu, H. Jin, W. Chen, S. Huo, X. Chen et al., Graphitized nitrogen-doped porous carbon composites derived from ZIF-8 as efficient microwave absorption materials. Mater. Res. Exp. **5**, 065602 (2018). https://doi.org/10.1088/2053-1591/aac67e

[S3] Y. Duan, Z. Xiao, X. Yan, Z. Gao, Y. Tang et al., Enhanced electromagnetic microwave absorption property of peapod-like MnO@carbon nanowires. ACS Appl. Mater. Interfaces **10**(46), 40078-40087 (2018). https://doi.org/10.1021/acsami.8b11395

[S4] M.A. Aslam, W. Ding, S. Rehman, A. Hassan, Y. Bian et al., Low cost 3D bio-carbon foams obtained from wheat straw with broadened bandwidth electromagnetic wave absorption performance. Appl. Surf. Sci. **543**, 148785 (2021). https://doi.org/10.1016/j.apsusc.2020.148785

[S5] C. Fu, D. He, Y. Wang, X. Zhao, Facile synthesis and microwave absorption performance of coated carbon nanotubes by porous Fe_3O_4@C nanorods. Synth. Met. **248**, 76-80 (2019). https://doi.org/10.1016/j.synthmet.2018.12.020

[S6] J. Tao, J. Zhou, Z. Yao, Z. Jiao, B. Wei et al., Multi-shell hollow porous carbon nanoparticles with excellent microwave absorption properties. Carbon **172**, 542-555 (2021). https://doi.org/10.1016/j.carbon.2020.10.062

[S7] J. Lv, X. Liang, G. Ji, B. Quan, W. Liu et al., Structural and carbonized design of 1D FeNi/C nanofibers with conductive network to optimize electromagnetic parameters

and absorption abilities. ACS Sustain. Chem. Eng. **6**(6), 7239-7249 (2018). https://doi.org/10.1021/acssuschemeng.7b03807

[S8] F. Wang, Y. Sun, D. Li, B. Zhong, Z. Wu et al., Microwave absorption properties of 3D cross-linked Fe/C porous nanofibers prepared by electrospinning. Carbon **134**, 264-273 (2018). https://doi.org/10.1016/j.carbon.2018.03.081

[S9] F. Meng, H. Wang, Wei, Z. Chen, T. Li et al., Generation of graphene-based aerogel microspheres for broadband and tunable high-performance microwave absorption by electrospinning-freeze drying process. Nano Res. **11**, 2847-2861 (2018). https://doi.org/10.1007/s12274-017-1915-6

[S10] H. Liu, Y. Li, M. Yuan, G. Sun, H. Li et al., In situ preparation of cobalt nanoparticles decorated in N-doped carbon nanofibers as excellent electromagnetic wave absorbers. ACS Appl. Mater. Interfaces **10**(26), 22591-22601 (2018). https://doi.org/10.1021/acsami.8b05211

[S11] J. Qiao, D. Xu, L. Lv, X. Zhang, F. Wang et al., Self-assembled ZnO/Co hybrid nanotubes prepared by electrospinning for lightweight and high-performance electromagnetic wave absorption. ACS Appl. Nano Mater. **1**(9), 5297-5306 (2018). https://doi.org/10.1021/acsanm.8b01303

[S12] I. Abdalla, A. Elhassan, J. Yu, Z. Li, B. Ding, A hybrid comprised of porous carbon nanofibers and rGO for efficient electromagnetic wave absorption. Carbon **157**, 703-713 (2020). https://doi.org/10.1016/j.carbon.2019.11.004

[S13] C. Han, M. Zhang, W.Q. Cao, M.S. Cao, Electrospinning and in-situ hierarchical thermal treatment to tailor C–$NiCo_2O_4$ nanofibers for tunable microwave absorption. Carbon **171**, 953-962 (2021). https://doi.org/10.1016/j.carbon.2020.09.067

[S14] X. Liang, B. Quan, J. Chen, W. Gu, B. Zhang et al., Nano bimetallic@carbon layer on porous carbon nanofibers with multiple interfaces for microwave absorption applications. ACS Appl. Nano Mater. **1**(10), 5712-5721 (2018). https://doi.org/10.1021/acsanm.8b01326

[S15] I. Abdalla, J. Shen, J. Yu, Z. Li, B. Ding, Co_3O_4/carbon composite nanofibrous membrane enabled high-efficiency electromagnetic wave absorption. Sci. Rep. **8**, 12402-12402 (2018). https://doi.org/10.1038/s41598-018-30871-2

[S16] X. Yuan, R. Wang, W. Huang, L. Kong, S. Guo et al., Morphology design of Co-electrospinning MnO-VN/C nanofibers for enhancing the microwave absorption performances. ACS Appl. Mater. Interfaces **12**(11), 13208-13216 (2020). https://doi.org/10.1021/acsami.9b23310

[S17] Y. Jiang, X. Fu, Z. Zhang, W. Du, P. Xie et al., Enhanced microwave absorption properties of Fe_3C/C nanofibers prepared by electrospinning. J. Alloys Compd. **804**, 305-313 (2019). https://doi.org/10.1016/j.jallcom.2019.07.038

[S18] Y. Wang, Y. Sun, Y. Zong, T. Zhu, L. Zhang et al., Carbon nanofibers supported by FeCo nanocrystals as difunctional magnetic/dielectric composites with broadband microwave absorption performance. J. Alloys Compd. **824**, 153980 (2020). https://doi.org/10.1016/j.jallcom.2020.153980

[S19] Y. Shen, Y. Wei, J. Li, Q. Li, J. Ma et al., Preparation of microwave absorbing Co-C nanofibers with robust superhydrophobic properties by electrospinning. J. Mater. Sci. Mater. Electron. **30**, 3365-3377 (2019). https://doi.org/10.1007/s10854-018-00610-4

[S20] X. Meng, S. Dong, Design and construction of lightweight C/Co heterojunction nanofibres for enhanced microwave absorption performance. J. Alloys Compd. **810**,

151806 (2019). https://doi.org/10.1016/j.jallcom.2019.151806

[S21] X. Huang, J. Zhang, M. Lai, T. Sang, Preparation and microwave absorption mechanisms of the NiZn ferrite nanofibers. J. Alloys Compd. **627**, 367-373 (2015). https://doi.org/10.1016/j.jallcom.2014.11.235

[S22] Y. Li, M. Yuan, H. Liu, G. Sun, In situ synthesis of $CoFe_2O_4$ nanocrystals decorated in mesoporous carbon nanofibers with enhanced electromagnetic performance. J. Alloys Compd. **826**, 154147 (2020). https://doi.org/10.1016/j.jallcom.2020.154147

[S23] C. Jin, Z. Wu, R. Zhang, X. Qian, H. Xu et al., 1D electromagnetic-gradient hierarchical carbon microtube via coaxial electrospinning design for enhanced microwave absorption. ACS Appl. Mater. Interfaces **13**(13), 15939-15949 (2021). https://doi.org/10.1021/acsami.1c03129

[S24] C. Zhang, Z. Wu, C. Xu, B. Yang, L. Wang et al., Hierarchical $Ti_3C_2T_x$ MXene/carbon nanotubes hollow microsphere with confined magnetic nanospheres for broadband microwave absorption. Small **18**(3), 2104380 (2022). https://doi.org/10.1002/smll.202104380

[S25] R. Han, W. Li, W. Pan, M. Zhu, D. Zhou et al., 1D magnetic materials of Fe_3O_4 and Fe with high performance of microwave absorption fabricated by electrospinning method. Sci. Rep. **4**, 7493 (2014). https://doi.org/10.1038/srep07493

[S26] X. Huang, J. Zhang, S. Xiao, T. Sang, G. Chen, Unique electromagnetic properties of the zinc ferrite nanofiber. Mater. Lett. **124**, 126-128 (2014). https://doi.org/10.1016/j.matlet.2014.03.049

[S27] Y. Hou, L. Cheng, Y. Zhang, Y. Yang, C. Deng et al., Electrospinning of Fe/SiC hybrid fibers for highly efficient microwave absorption. ACS Appl. Mater. Interfaces **9**(8), 7265-7271 (2017). https://doi.org/10.1021/acsami.6b15721

[S28] R. Guo, D. Su, F. Chen, Y. Cheng, X. Wang et al., Hollow beaded Fe_3C/N-doped carbon fibers toward broadband microwave absorption. ACS Appl. Mater. Interfaces **14**(2), 3084-3094 (2022). https://doi.org/10.1021/acsami.1c21272

[S29] R. Guo, D. Su, K. Zou, C. Zhang, F. Cen et al., N-doped carbon fibers with embedded ZnFe and Fe_3C nanoparticles for microwave absorption. ACS Appl. Nano Mater. **4**(10), 11070-11079 (2021). https://doi.org/10.1021/acsanm.1c02532

[S30] J. Qiao, X. Zhang, D. Xu, L. Kong, L. Lv et al., Design and synthesis of TiO_2/Co/carbon nanofibers with tunable and efficient electromagnetic absorption. Chem. Eng. J. **380**, 122591 (2020). https://doi.org/10.1016/j.cej.2019.122591

[S31] M. Yang, Y. Yuan, Y. Li, X. Sun, S. Wang et al., Dramatically enhanced electromagnetic wave absorption of hierarchical CNT/Co/C fiber derived from cotton and metal-organic-framework. Carbon **161**, 517-527 (2020). https://doi.org/10.1016/j.carbon.2020.01.073

[S32] Z. Zhao, K. Kou, H. Wu, 2-methylimidazole-mediated hierarchical Co_3O_4/N-doped carbon/short-carbon-fiber composite as high-performance electromagnetic wave absorber. J. Colloid Interface Sci. **574**, 1-10 (2020). https://doi.org/10.1016/j.jcis.2020.04.037

[S33] D. Liu, R. Qiang, Y. Du, Y. Wang, C. Tian et al., Prussian blue analogues derived magnetic FeCo alloy/carbon composites with tunable chemical composition and enhanced microwave absorption. J. Colloid Interface Sci. **514**, 10-20 (2018). https://doi.org/10.1016/j.jcis.2017.12.013

[S34] X. Li, J. Feng, Y. Du, J. Bai, H. Fan et al., One-pot synthesis of $CoFe_2O_4$/graphene oxide hybrids and their conversion into FeCo/graphene hybrids for lightweight and highly efficient microwave absorber. J. Mater. Chem. A **3**, 5535-5546 (2015). https://doi.org/10.1039/C4TA05718J

[S35] G. Li, L. Wang, W. Li, Y. Xu, Fe-, Co-, and Ni-loaded porous activated carbon balls as lightweight microwave absorbents. ChemPhysChem **16**, 3458-3467 (2015). https://doi.org/10.1002/cphc.201500608

[S36] X. Zeng, B. Yang, L. Zhu, H. Yang, R. Yu, Structure evolution of Prussian blue analogues to CoFe@C core–shell nanocomposites with good microwave absorbing performances. RSC Adv. **6**(107), 105644-105652 (2016). https://doi.org/10.1039/C6RA18928H

[S37] W. Chu, Y. Wang, Y. Du, R. Qiang, C. Tian et al., FeCo alloy nanoparticles supported on ordered mesoporous carbon for enhanced microwave absorption. J. Mater. Sci. **52**, 13636-13649 (2017). https://doi.org/10.1007/s10853-017-1439-1

[S38] C. Wang, T. Xu, C.A. Wang, Microwave absorption properties of C/(C@CoFe) hierarchical core–shell spheres synthesized by using colloidal carbon spheres as templates. Ceram. Int. **42**, 9178-9182 (2016). https://doi.org/10.1016/j.ceramint.2016.03.012

[S39] Y. Zhang, M. Yao, C. Liu, H. Zhao, X. Miao et al., Reduced graphene oxide-$CoFe_2O_4$/FeCo nanoparticle composites for electromagnetic wave absorption. ACS Appl. Nano Mater. **3**(9), 8939-8948 (2020). https://doi.org/10.1021/acsanm.0c01693

[S40] G. Guan, G. Gao, J. Xiang, J. Yang, L. Gong et al., $CoFe_2$/$BaTiO_3$ hybrid nanofibers for microwave absorption. ACS Appl. Nano Mater. **3**(8), 8424-8437 (2020). https://doi.org/10.1021/acsanm.0c01855

[S41] Y. Guo, D. Wang, J. Wang, Y. Tian, H. Liu et al., Hierarchical HCF@NC/Co derived from hollow loofah fiber anchored with metal–organic frameworks for highly efficient microwave absorption. ACS Appl. Mater. Interfaces **14**(1), 2038-2050 (2022). https://doi.org/10.1021/acsami.1c21396
